# Inteligência Artificial em Cardiologia: Conceitos, Ferramentas e Desafios – “Quem Corre é o Cavalo, Você Precisa ser o Jóquei”

**DOI:** 10.36660/abc.20180431

**Published:** 2020-05-12

**Authors:** Erito Marques de Souza, Fernando de Amorim Fernandes, Celine Lacerda de Abreu Soares, Flavio Luiz Seixas, Alair Augusto Sarmet M.D. dos Santos, Ronaldo Altenburg Gismondi, Evandro Tinoco Mesquita, Claudio Tinoco Mesquita

**Affiliations:** 1 Universidade Federal Fluminense Niterói RJ Brasil Universidade Federal Fluminense, Niterói, RJ – Brasil; 2 Universidade Federal Rural do Rio de Janeiro Departamento de Tecnologias e Linguagens Nova Iguaçu RJ Brasil Universidade Federal Rural do Rio de Janeiro - Departamento de Tecnologias e Linguagens, Nova Iguaçu, RJ – Brasil

**Keywords:** Inteligência Artificial/tendências, Sistemas de Computação/tendências, Aprendizado de Máquina/tendências, Doenças Cardiovasculares, Tomada de Decisão Clínica

## Abstract

Os recentes avanços ao nível de hardware e a crescente exigência de personalização dos cuidados associados às necessidades urgentes de criação de valor para os pacientes contribuíram para que a Inteligência Artificial (IA) promovesse uma mudança significativa de paradigma nas mais diversas áreas do conhecimento médico, em particular em Cardiologia, por sua capacidade de apoiar a tomada de decisões e melhorar o desempenho diagnóstico e prognóstico. Nesse contexto, o presente trabalho faz uma revisão não-sistemática dos principais trabalhos publicados sobre IA em Cardiologia, com foco em suas principais aplicações, possíveis impactos e desafios.

## Introdução

A vida cotidiana de uma pessoa exige uma enorme quantidade de conhecimento sobre o mundo e o volume de dados em saúde cresce exponencialmente em todo o mundo.^1^ Por outro lado, o conhecimento biomédico está sempre se expandindo de maneira ativa e dinâmica e não pode ser processado ou armazenado por um único cérebro humano. Essa situação torna muito difícil para o médico contemporâneo manter-se atualizado com um amplo espectro de novos dados e descobertas, bem como em relação à utilização dessas informações com facilidade e em tempo hábil.^2^ Acrescentam-se a esse quadro as significativas taxas de *burnout* entre profissionais de saúde^3 , 4^ e o importante impacto de erros médicos – que nos Estados Unidos representam a terceira principal causa de morte.^5^ Esse panorama traz consigo a necessidade de reorganizar a estrutura produtiva dos serviços de saúde, associada a vários desafios e novas perspectivas. Dado que o sistema de saúde atual é geralmente improdutivo e /ou caro, é imperativo desenvolver estratégias alternativas e inovadoras. O foco central para alcançar esse objetivo deve ser aumentar o valor para o paciente – resultados alcançados por dólar gasto – para que bons resultados, obtidos com eficiência, sejam um objetivo a ser perseguido.^6^

Além disso, os recentes avanços ao nível de hardware relacionados ao processamento paralelo, a existência de vários métodos de aprendizado de máquina e a enorme quantidade de dados anotados contribuíram para que a inteligência artificial (IA) promovesse uma mudança significativa de paradigma nas mais diversas áreas do conhecimento médico, e particularmente em Cardiologia, por sua capacidade de apoiar a tomada de decisões que podem melhorar o desempenho diagnóstico e prognóstico. Esses impactos devem ser avaliados na perspectiva da segurança do paciente, personalização dos cuidados, criação de valor para os pacientes, no âmbito da vigilância tecnológica – que gradualmente consolida a IA como fundamental para uma prática médica de excelência.^7 - 11^

**Figura 1:**
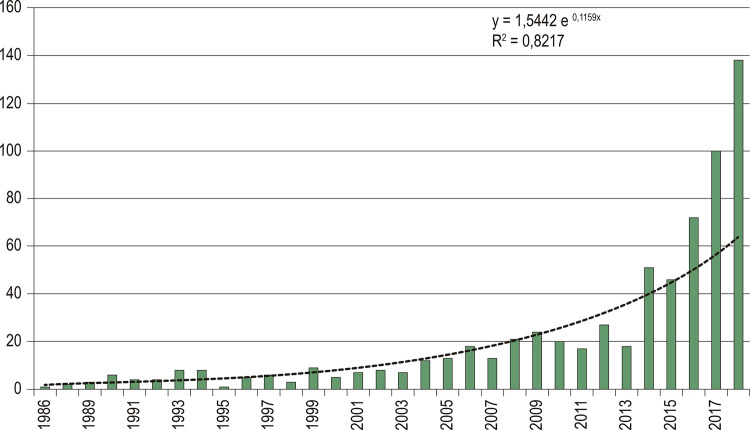
– *Evolução do número de trabalhos relacionados (Inteligência Artificial ou Aprendizado de Máquina, ou Aprendizado Automático) e Cardiologia. Fonte: Pubmed. Acessado em 15/12/2018. Palavras do Medical Subject Headings (MeSH): Cardiologia e Aprendizado de Máquina.*

Esse cenário faz com que a IA, dada sua importância, seja considerada por muitos como a nova eletricidade. As principais Revistas de Cardiologia publicaram revisões nessa área e o número de artigos sobre o assunto segue uma tendência crescente, como mostra a Figura 1 – esse comportamento também é observado em outras especialidades médicas, como a neurologia. Portanto, o presente trabalho faz uma revisão não-sistemática dos principais trabalhos publicados sobre IA em Cardiologia, com foco em suas principais aplicações, potenciais impactos e desafios. A próxima seção apresenta os fundamentos conceituais do tópico, seguidos do motivo pelo qual a cardiologia precisa da IA e de suas principais ferramentas. Por fim, são apresentados os principais desafios, perspectivas e conclusões.

### O que é inteligência artificial (IA)?

O termo AI foi utilizado pela primeira vez na Conferência de Dartmouth em 1956.^12^ No entanto, a possibilidade de que as máquinas fossem capazes de simular o comportamento humano e realmente pensar foi levantada anteriormente por Alan Turing em 1950, que desenvolveu um teste para diferenciar os seres humanos de máquinas – denominado teste de Turing.^13^

Basicamente, a IA é o produto da combinação de modelos matemáticos sofisticados e computação, que permite o desenvolvimento de algoritmos complexos capazes de emular a inteligência humana. Todo esse processo inicia-se com a construção de um banco de dados representativo do problema que se deseja estudar – coletado e processado adequadamente – chamados dados saudáveis. Essa etapa é de fundamental importância, pois os algoritmos provavelmente não terão um bom desempenho se esse pré-requisito não for obtido: “ *lixo na entrada, lixo na saída”* .

A natureza desses dados é bastante variada, variando de dados socioambientais, clínico-laboratoriais a dados ômicos (por exemplo, metaboloma, proteoma, epigenoma, lipidoma) e informações das intensidades de vermelho, verde e azul (sistema RGB) de cada pixel que compõe uma imagem, por exemplo. Fontes igualmente diversificadas de tais dados incluem aquelas obtidas de registros médicos eletrônicos ou mesmo dispositivos “ *wearable* ” (“vestíveis”). Nesse contexto, o termo “Big Data” é utilizado para descrever uma enorme coleção de dados para os quais os métodos tradicionais de análise não são bem-sucedidos na análise, pesquisa, interpretação e armazenamento.^9^

Destacamos o uso dessas ferramentas em problemas de classificação, regressão e clusterização. Depois de obter dados saudáveis e construir o banco de dados, é importante avaliar quais modelos matemáticos de IA são mais apropriados para o problema que se deseja resolver. Em seguida, os modelos escolhidos devem ser implementados utilizando alguma linguagem de programação.

**Figura 2:**
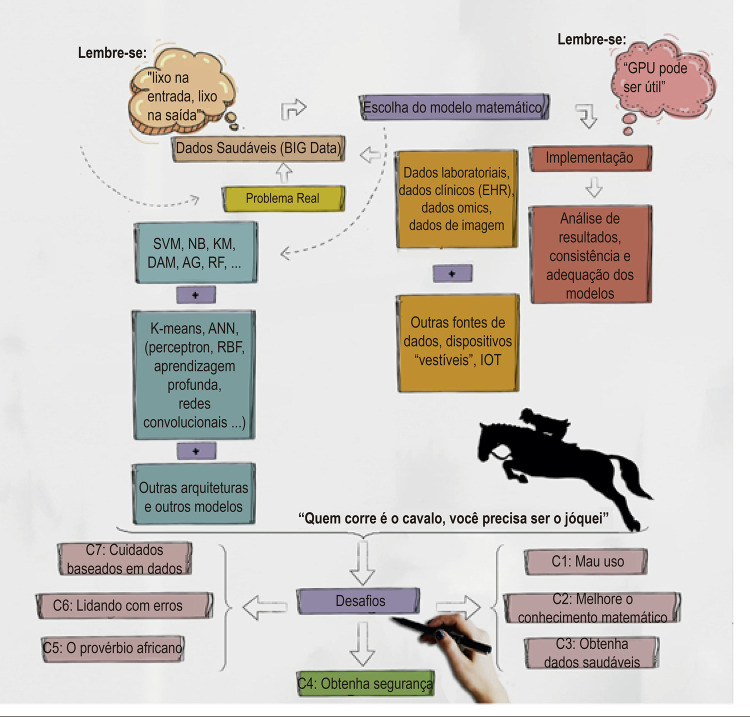
– *Ilustração principal.*

Uma combinação de modelos também pode ser útil. Os resultados obtidos pelo algoritmo devem ser analisados em termos de coerência e adequação. Essas etapas estão resumidas na Figura 2 .

### Por que a cardiologia precisa de inteligência artificial?

O desenvolvimento de algoritmos de IA tem a vantagem de não exigir muitas suposições em relação aos dados subjacentes.^8^ Outro ponto é que a natureza desses modelos matemático-computacionais permite, a partir de dados observacionais, um alto nível de evidência devido ao seu alto desempenho, o que certamente representa uma mudança significativa de paradigma na medicina baseada em evidências. Deve-se notar que os ensaios clínicos tradicionais são geralmente lentos, caros, demorados e limitados em tamanho.^14^ Além disso, quando o banco de dados é alimentado com mais dados (saudáveis), em geral, há uma melhoria no desempenho dos algoritmos – o que permite que os estudos tenham um caráter contínuo ao longo do tempo.

Esse novo arquétipo pode guiar a alocação de recursos escassos na área da saúde e facilitar a identificação eficiente e precisa de decisões que favoreçam a individualização do cuidado com base no fluxo de informações que emergem de um ecossistema integrado e complexo: é uma medicina de precisão.^15 , 16^ Portanto, pode-se inferir que a prática das ciências cardiovasculares terá impactos significativos, que se traduzirão em uma abordagem personalizada e melhores resultados.

### Conceitos básicos em inteligência artificial

Um banco de dados genérico pode ser organizado em uma matriz de linhas e colunas. Cada linha indica um elemento de um conjunto de objetos a ser avaliado de acordo com as mesmas características. Cada coluna, por sua vez, expressa os valores de um determinado atributo para as várias linhas no banco de dados e cada linha representa uma lição a ser aprendida pelo modelo matemático-computacional. Dessa maneira, o termo Aprendizado de Máquina (ML, *machine learning* ) traz consigo a possibilidade de “aprender” a partir de um conjunto de lições. O termo IA é frequentemente utilizado de forma intercambiável com o termo ML. No entanto, ML é um subconjunto de algoritmos de IA relacionados à capacidade de aprender a partir de uma grande quantidade de dados. A IA é mais ampla e engloba a execução de tarefas que normalmente estão relacionadas à inteligência humana, tais como reconhecimento de padrões, resolução de problemas, compreensão de linguagem ou reconhecimento de objetos e sons.^17^

Costuma-se dizer que os tipos de aprendizado podem ser:

- **Supervisionados:** quando o algoritmo recebe informações sobre cada lição, bem como os rótulos associados a ela, tendo um papel importante em relação à previsão. Por exemplo, se é desejado prever se um paciente é mais suscetível à tosse com o uso de inibidores da enzima de conversão da angiotensina, a análise deve ser realizada baseada em um banco de dados saudáveis contendo um grupo de pacientes que demonstrou essa reação e em outro grupo no qual esse fato não foi observado.
- **Não-supervisionado:** quando os rótulos das lições não são fornecidos *a priori* , cabe ao algoritmo encontrar estruturas ocultas no banco de dados. Um exemplo hipotético é a clusterização de um banco de dados de pacientes com cardiomiopatia hipertrófica de acordo com os achados de exame de imagem.
- **Reforço:** inspirado na biologia comportamental, é um tipo de aprendizado baseado em recompensa.^18 , 19^

Outro conceito importante é o da computação cognitiva. Ele pode ser entendido como um conjunto de sistemas de autoaprendizagem destinados a imitar o processo de pensamento humano com base no uso de ferramentas de ML, reconhecimento de padrões e processamento natural de linguagem.^9^ O IBM Watson é um exemplo da computação cognitiva na área médica.^20 , 21^

### Algumas ferramentas e aplicativos de inteligência artificial

Atualmente, existem vários modelos de ML, cada um deles com diversas particularidades, usos e limitações variados. As aplicações de alguns desses modelos em Cardiologia são explicadas nos parágrafos seguintes, enquanto uma breve descrição de cada um deles e seu tipo é mostrada na Tabela 1 .

**Tabela 1:** – Breve descrição e classificação das principais ferramentas de ML

Ferramenta	Descrição	Aprendizado
SVM	É útil para problemas de classificação de dois grupos. A ideia é encontrar uma função chamada hiperplano a partir da resolução de um sistema linear construído a partir das várias lições do subconjunto de treinamento. ^40^ Esse hiperplano é utilizado para agrupar as lições do subconjunto de teste em dois grupos disjuntos.	Supervisionado
NB	Foi inspirado nos estudos do reverendo Bayes sobre a probabilidade condicional. ^41^ Essas probabilidades são usadas para identificar a categoria (de um total de n possível) a que uma lição específica pertence. ^42^	Supervisionado
KNN	Diz-se que uma norma de vetor é uma função matemática, que satisfaz propriedades específicas, e associa um vetor a um valor maior ou igual a zero. ^43^ A norma da diferença entre dois vetores é a distância entre eles. O KNN utiliza uma norma para calcular a distância entre todos os vetores (lições) que compõem o banco de dados. Então, para cada vetor do banco de dados, os vetores k mais próximos a ele são determinados. A inclusão em um determinado grupo é obtida a partir de um sistema de votação majoritária entre os vizinhos. ^44^^,^^45^	Supervisionado
AG	Algoritmos inspirados na evolução biológica das espécies, nos quais cada possível candidato para solucionar o problema é modelado como um cromossomo constituído por um conjunto de genes, que durante a execução do algoritmo é submetido a operações de cruzamento e mutação para obter melhor soluções que as atuais. ^46^ Dessa forma, eles permitem que um banco de dados seja separado, por exemplo, em dois grupos distintos – que possuem ou não uma característica específica.	Supervisionado
RF	Esse método é baseado na construção de várias árvores de decisão. O primeiro passo é obter várias amostras aleatórias (com reposição) de lições para criar outros bancos de dados, um processo chamado “bootstrapping”. Cada um desses novos bancos de dados dará origem a uma árvore de decisão, obtida de forma iterativa, a partir de um subconjunto de variáveis (características). Após a construção de todas as árvores, uma nova lição no banco de dados deve ser alocada ao grupo que tem o maior número de árvores de decisão, mostrando que ele pertence a esse grupo (maioria dos votos). ^47^^,^^48^	Supervisionado
K-means	Permite particionar um banco de dados em k grupos com características semelhantes. Para isso, é necessário atualizar, de maneira iterativa, um conjunto de vetores, chamados centroides de referência de cada grupo e calcular a distância de cada lição para cada um. Uma lição é sempre alocada ao centroide para o qual ela tem a menor distância. O gráfico de cotovelo é geralmente utilizado para determinar o número ideal de grupos a serem separados do banco de dados. ^49^	Não supervisionado
ANN	Inspirado nos sistemas nervosos biológicos, é utilizada uma estrutura chamada gráfico – um conjunto de nós e bordas – em que os nós são estratificados e conectados por bordas com valores, que representam um peso atribuído a uma determinada conexão. A ideia é que, a partir de um conjunto de entradas, esses pesos sejam utilizados corretamente para produzir uma saída. Várias arquiteturas foram propostas para redes neurais, desde as mais simples, como a perceptron, até as mais sofisticadas, como a função de base radial, redes convolucionais e aprendizado profundo. No aprendizado profundo, além das camadas de entrada e saída, existem camadas ocultas que aumentam significativamente o número de pesos a serem atualizados e geralmente exigem grandes esforços computacionais. A rede convolucional é um tipo de aprendizado profundo inspirada no córtex visual de animais que tem um papel importante na análise de imagens. Autoencoders e redes neurais de Kohonen são exemplos de aprendizado não supervisionado. ^1^^,^^7^^,^^50^^-^^52^	Não supervisionado ou Supervisionado
GB	É um método baseado em árvore que utiliza gradientes, vetores relacionados à direção do aumento máximo em uma função matemática, para produzir árvores de decisão sequenciais a serem combinadas para aprimorar a previsão. Variantes desta abordagem incluem o Stochastic Gradient Descent que incorpora uma sub-amostra aleatória para GB. ^53^^,^^54^	Supervisionado

- ***Support Vector Machine*** **(SVM):** utilizada por Samad et al.,^22^ para prever com sucesso a deterioração da função ventricular em pacientes submetidos a reparo da tetralogia de Fallot a partir de um banco de dados de 153 pacientes com dados clínicos, eletrocardiográficos e de ressonância magnética cardíaca. Em relação à previsão de qualquer deterioração (menor ou maior) vs. nenhuma deterioração, a média da área sob a curva (AUC) foi de 0,82 ± 0,06.^22^ Berikol et al.^23^ utilizaram dados clínicos, laboratoriais (níveis de troponina I e CK-MB), do ECG e ecocardiográficos de 228 pacientes que apresentaram dor no peito no pronto-socorro para classificação quanto à presença ou ausência de Síndrome Coronariana Aguda. Precisão, sensibilidade e especificidade foram, respectivamente, 99,19, 98,22 e 100%.^23^ Betancur et al.,^24^ também utilizaram o SVM para definir com maior precisão o posicionamento do plano da válvula (PV) mitral durante a segmentação ventricular esquerda nos exames de Tomografia Computadorizada por Emissão de Fóton Único (SPECT). Imagens de 392 pacientes foram analisadas e os bons resultados obtidos foram compatíveis com a opinião de especialistas da área – AUC: 0,82 [0,74-0,9] para detecção regional de áreas de estenose obstrutiva e áreas de déficit de perfusão total isquêmica.^24^
- ***Naive Bayes*** **(NB):** Paredes et al.,^25^ utilizaram uma fusão de NB e algoritmo genético para prever o risco de ocorrência de eventos cardiovasculares (por exemplo, hospitalização ou morte), com base em dados de 559 pacientes com Síndrome Coronariana Aguda - Infarto do Miocárdio sem Supradesnivelamento do Segmento ST (SCA-IAMSST). Sensibilidade e especificidade foram, respectivamente, 79,8, e 83,8.^25^
- ***K-nearest neighbors*** **(KNN):** Al-Mallah et al.,^26^ compararam a previsão de mortalidade por todas as causas em 10 anos entre o modelo de regressão logística clássico e o KNN, considerando um banco de dados de 34.212 pacientes com informações clínicas e informações obtidas após o teste de esforço em esteira utilizando o protocolo padrão de Bruce.^26^ Os resultados obtidos por essa ferramenta de ML mostraram sensibilidade de 87,4% e especificidade de 97,2%, melhores do que o desempenho preditivo do tradicional escore de risco *Atherosclerosis Cardiovascular Disease Risk Score* (ASCVD).
- ***Genetic algorithms*** **(GA):** Smisek et al.,^27^ desenvolveram um dispositivo *wearable* (“vestível”) para detectar arritmias a partir do registro de informações de um eletrocardiograma de derivação única. Os dados foram analisados a partir de uma combinação do (SVM), árvore de decisão e regras baseadas em limiares. Algoritmos genéticos foram usados para selecionar as características mais adequadas a serem utilizadas no trabalho. Em relação à detecção de fibrilação atrial, obteve-se um escore F1 (média harmônica de valor preditivo positivo e sensibilidade) de 0,81.^27^ Stuckey et al.,^28^ utilizaram a Análise de Tomografia Espacial de Fase Cardíaca – um método pioneiro que dispensa o uso de radiação e contraste, bem como a realização de testes de esforço ou farmacológico – combinado com modelos de ML (por exemplo, algoritmos genéticos) para analisar os sinais da fase torácica. Neste estudo, os autores utilizaram essa ferramenta para avaliar pacientes com doença coronariana e dor torácica que foram encaminhados pelo médico para realizar uma angiografia. Foram estudados 606 pacientes e os resultados mostraram sensibilidade de 92%, especificidade de 62% e valor preditivo de 96% para doença coronariana.^28^
- ***Random Forests*** **(RF):** Samad et al.,^29^ analisaram um banco de dados composto por variáveis clínicas e eletrocardiográficas para avaliar a sobrevida em 10 diferentes períodos de tempo (variando de 6 a 60 meses), considerando um total de 171.510 pacientes. A RF foi utilizada, com excelentes resultados, melhor do que aqueles obtidos por meio de escores tradicionais, como o escore de risco de Framingham e o escore das diretrizes da ACC/AHA. A área sob a curva (AUC) foi superior a 0,82.^29^ Ambale-Venkatesh, et al.,^30^ utilizaram informações de testes não invasivos, questionários, biomarcadores e exames de imagem de 6.814 pacientes para construir 739 variáveis (características), a fim de aplicar uma variante do RF – chamada *survivor random forests* ^31^ – para prever eventos cardiovasculares. (morte por todas as causas, acidente vascular cerebral, todas as doenças cardiovasculares, doença coronariana, fibrilação atrial e insuficiência cardíaca), que apresentou um desempenho melhor do que os escores de risco estabelecidos, como por exemplo, MESA-CHD, AHA/ASCVD e o escore de Framingham, com maior acurácia na previsão (diminuição de 10%-25% do escore de Brier)^30 , 31^ .
- ***K-means*** **:** Cikes et al.,^32^ utilizaram um banco de dados composto por variáveis clínicas e parâmetros ecocardiográficos para os quais foram aplicados dois modelos de ML, *K-means* e *Multiple Kernel Learning* , a fim de categorizar os pacientes em grupos mutuamente exclusivos para avaliar a resposta à terapia de ressincronização cardíaca. Foram analisados 1.106 pacientes e identificados quatro grupos disjuntos, dois deles com a melhor resposta à terapia.^32^
- ***Artificial Neural Networks*** **(ANN):** Kwon et al.,^33^ em um estudo multicêntrico com 52.131 pacientes, construíram um sistema de alerta precoce baseado em aprendizado profundo capaz de prever a ocorrência de parada cardíaca em um hospital. O modelo demonstrou alto desempenho quando comparado aos sistemas tradicionais do tipo “ *track-and-trigger* ” (rastreamento e disparo). A área sob a curva foi de 0,82.^33^ Rubin et al.,^34^ tiveram resultados preliminares promissores com o uso de redes neurais com arquitetura convolucional para avaliar sinais eletrocardiográficos e classificá-los em fibrilação atrial, ritmo sinusal (normal) ou ruído – o escore F1 alcançado foi de 0,82.^34^ Zhang et al.,^35^ também utilizaram redes neurais convolucionais para analisar um banco de dados com 14.035 exames ecocardiográficos para detectar a presença de doenças como cardiomiopatia hipertrófica, amiloidose cardíaca e hipertensão arterial pulmonar com alto desempenho: as estatísticas C foram respectivamente de 0,93, 0,87 e 0,85.^35^ Nakajima et al.,^36^ utilizaram uma ANN para avaliar a presença de doença coronariana após a realização de cintilografia do miocárdio. Os resultados foram obtidos com alta precisão e desempenho superior aos escores tradicionais utilizados. Por exemplo, a AUC para pacientes com infarto do miocárdio antigo com base em defeitos na fase de repouso foi de 0,97.^36^
- ***Gradient Boosting*** **(GB):** Mortazavi et al.,^37^ utilizaram o GB para predizer o risco de sangramento após intervenção coronária percutânea e demonstraram que essas ferramentas podem ajudar a identificar pacientes que se beneficiariam de estratégias objetivando a redução do risco de sangramento. Foram analisados 3.316.465 procedimentos e obtida uma estatística C de 0,82^37^ . Hernesniemi et al.,^38^ também propuseram um GB para prever a mortalidade na síndrome coronariana aguda, analisando 9.066 pacientes consecutivos. A AUC foi de 0,89 e o modelo apresentou melhor desempenho do que o do escore tradicional GRACE.^38^

É importante observar que, ao utilizar qualquer modelo de ML, deve-se ter em mente um grande problema que pode surgir, chamado sobreajuste ( *overfitting* ). Ele ocorre quando um modelo descreve muito bem os exemplos (subconjunto de treinamento) e apresenta baixo desempenho quando aplicado a outras instâncias do mesmo fenômeno.^39^ Além disso, é importante dizer que não há resultado teórico que garanta que qualquer um dos algoritmos de IA seja melhor que os outros em qualquer aplicação.

Portanto, essa escolha depende de diversas variáveis, como a natureza do problema em análise, o tempo e os recursos disponíveis para resolvê-lo. A combinação de técnicas que geram modelos híbridos também pode ser de grande valor. Por outro lado, o uso de ferramentas para processamento paralelo, como as GPUs ( *Graphic Processing Units* ), tem sido de grande valia para melhorar o desempenho dos modelos de ML, principalmente em relação ao tempo computacional necessário para executá-los.

### Desafios e perspectivas futuras

Como destacado anteriormente, as aplicações de IA em cardiologia aumentaram bastante nos últimos anos e seu potencial de crescimento é enorme. No entanto, esse cenário traz consigo a necessidade de superar alguns desafios, tais como: limites éticos de uso (uso indevido), aprimoramento do conhecimento matemático, aquisição de dados saudáveis, desenvolvimento de segurança, necessidade de colaboração, atenção a erros e cuidados baseados em dados. Tudo isso é discutido abaixo e está resumido na Figura 2 .

- **Desafio 1 –** limites éticos de uso (uso indevido): como toda tecnologia disruptiva, os limites da ética precisam ser repensados e amplamente discutidos. Os algoritmos de ML podem ser mal utilizados e enganosos. Como exemplo, uma obra de grande repercussão foi publicada por Wang e Kosinski (2018). Os autores usaram aprendizado profundo e obtiveram resultados expressivos para prever se um indivíduo é gay ou não em um banco de dados de imagens dos rostos dos participantes do estudo.^55^ De maneira similar, os mesmos algoritmos de IA podem ser utilizados para detectar, por exemplo, se um paciente desenvolverá ou não fibrilação atrial ou qualquer cardiomiopatia futura. Essas informações poderiam ser utilizadas pelas empresas para aumentar os valores de seus planos de saúde ou até mesmo negar a associação ao plano devido a um alto custo? E se for detectado que um bebê nascerá com doença cardíaca congênita devido à análise dos dados genéticos, clínico-laboratoriais e de imagem (ou outros) de seus pais? Isso poderia abrir espaço para um tipo de neoeugenia. Esse debate ganhou uma ênfase adicional com o surgimento da técnica CRISP-Cas9, que permite a edição do DNA.^56^ Nesse contexto, ao estimular um debate com a sociedade sobre o assunto, a transparência e a regulamentação são pilares fundamentais a serem preservados.
- **Desafio 2 –** melhorar o conhecimento de matemática: o advento desse novo tipo de ser humano inacreditável ( *Homo incredibile* ), que apoia suas decisões em dados, carrega consigo o papel fundamental da matemática e da computação nesta revolução atualmente em andamento. Essa revolução trará possibilidades inimagináveis na prática médica, como a construção de *phenomappings* de qualidade – modelos de ML desenvolvidos com o objetivo de agrupar pacientes em função de sua grande massa de características fenotípicas, a fim de facilitar o processo de tomada de decisão.^57^ Assim, é necessário que essas competências sejam estimuladas precocemente, principalmente com foco na solução de problemas relacionados à realidade para a qual se deseja promover melhorias. Isso certamente se refletirá na necessidade de reformular o conteúdo cardiovascular (e por que não dizer, o conteúdo médico em geral) dos cursos de graduação e pós-graduação em Medicina: uma educação passiva ou meramente expositiva, com uma carga extensa e priorizando a capacidade de memória do aluno, parece cada vez mais inadequada, pois se percebe que a Medicina deve ser um espaço de criatividade e geração de valor.
- **Desafio 3 –** obtenha dados saudáveis: o uso de dados saudáveis é de valor fundamental para o sucesso dos algoritmos. Assim, é necessário que as unidades de saúde incentivem seus profissionais de saúde a respeito do rigor no nível de preenchimento/obtenção de dados, bem como a manutenção de qualquer fonte de dados, de formulários, prontuários eletrônicos, dados de imagem ou mesmo dados não convencionais, como os obtidos por Medina et al.^58^ – que desenvolveram uma ferramenta de saúde de redes sociais online ( *Online Social Networks Health* ) bem-sucedida, na qual o próprio paciente insere anonimamente informações de monitoramento de saúde, incluindo dados fisiológicos, atividades diárias, estados emocionais e interação com outros pacientes.^58^ Portanto, o gerenciamento de dados se torna tão importante quanto outros comportamentos de rotina na medicina baseada em evidências, como lavagem adequada das mãos ou até o uso de um desfibrilador durante uma parada cardíaca. Dessa maneira, a formação de equipes de dados multidisciplinares e o treinamento constante das equipes assumem um papel primordial. Vale ressaltar que grande parte da lentidão e dificuldade que algumas unidades de saúde têm no uso de modelos de ML está ligada a dados saudáveis ausentes ou incipientes.
- **Desafio 4 –** obtenha segurança: o advento dessas ferramentas traz consigo uma preocupação fundamental com a segurança dos dados, a um nível nunca experimentado, pois o acesso a esses dados por pessoas não autorizadas pode levar a consequências catastróficas para as instituições de saúde e para os pacientes. A criação de uma equipe de segurança desempenha um papel importante nesse novo processo. O Regulamento Geral de Proteção de Dados ( *General Data Protection Regulation* ) representa um avanço nessa direção. *Blockchain* e suas variantes são ferramentas importantes que podem melhorar substancialmente a segurança.
- **Desafio 5 –** necessidade de colaboração (o provérbio Africano): há um provérbio africano que diz: “ *se você quer ir rápido, vá sozinho, mas se quiser ir longe, vá com muitos* ”. Isso se aplica muito a esse ambiente de dados: a colaboração entre instituições permite a construção de enormes bancos de dados saudáveis ( *Big Data* ), o que tende a favorecer o desempenho dos algoritmos de ML.
- **Desafio 6 –** lidando com erros: Uma questão importante diz respeito aos erros dos modelos de IA. É inadequado acreditar que esses modelos estão livres de erros. Por exemplo, pode ser o resultado de sobreajuste ou da ocorrência de dados não íntegros – que tornam os resultados não confiáveis. No entanto, a prática mostrou alto desempenho em várias aplicações. Esses modelos são probabilísticos e é sempre desejável que seus erros sejam mínimos. Esse cenário tem implicações clínicas, por exemplo, um modelo de IA que prevê com 99% de probabilidade que um paciente tem maior propensão do que a população em geral a ter miocardite cardíaca ou amiloidose. Há uma probabilidade, embora pequena, de que isso não ocorra e de que o procedimento adotado pelo cardiologista seja inadequado. Nesse caso, a questão é quem pode ser responsabilizado nesses casos? É apropriado? O paciente deve assinar um termo de consentimento nesses casos? Certamente, a solução inclui uma regulamentação robusta do uso dessas ferramentas e o fortalecimento de um novo tipo de relacionamento: médico-paciente-dados.
- **Desafio 7 –** gestão de cuidados baseados em dados: enquanto as ferramentas de ML seguem um caminho inexorável, por outro lado, vários profissionais de saúde continuam com medo dessas ferramentas devido a sua possível capacidade de substituir os médicos em suas tarefas. Entretanto, quando a história da Medicina é lembrada, vale ressaltar, por exemplo, que o aparecimento de máquinas automatizadas para realizar o hemograma não substituiu o hematologista, mas resultou em uma maior velocidade do processo de trabalho e permitiu ao profissional atuar em outras questões importantes da especialidade. A ideia central é fornecer melhor suporte para a tomada de decisão, incluindo melhor desempenho. Trata-se da gestão assistencial baseada em dados, com alto dinamismo e atualização constante – o que promoverá maior personalização do cuidado^59^ e avaliação em tempo real da experiência dos usuários do sistema de saúde, visando gerar valor para o paciente. Nesse contexto, as tarefas mecânicas serão substituíveis e uma diversidade de novas tarefas será incluída na rotina do cardiologista de precisão, desde a construção adequada das bases de dados até a reflexão crítica sobre os resultados obtidos pelos modelos matemático-computacionais, bem como o desenvolvimento de um relacionamento médico-paciente-dados adequado. Portanto, há uma migração de habilidades humanas, bem como a expansão de suas capacidades a partir do surgimento de novas ferramentas, que devem fazer parte do arsenal técnico do cardiologista do século XXI. Esse panorama nos permite comparar os modelos de ML a um cavalo e os médicos a jóqueis: “ *quem corre é o cavalo, você precisa ser o jóquei* ”.

## Conclusões

A IA, de fato, tem se mostrado uma ferramenta fundamental para a prática clínica da cardiologia atual. Diversas aplicações foram realizadas com sucesso e permitiram melhorias significativas do ponto de vista diagnóstico e terapêutico e em relação ao atendimento personalizado. Para poder utilizar tais ferramentas, é imperativo que dados saudáveis sejam utilizados, o que certamente implica um novo design no *modus operandi* de muitos serviços de saúde. A natureza desses dados é variada e inclui novas fontes, como dispositivos “vestíveis” e dados *omic* . Por outro lado, esse novo ecossistema digital requer uma aquisição de conhecimento que não é tradicionalmente encontrado em cursos regulares de medicina. Portanto, um redesenho curricular é necessário e deve ser objeto de um profundo debate e ações específicas.

Por outro lado, toda a panaceia trazida pela IA não está isenta de desafios, como os limites éticos de seu uso, a necessidade de aprimorar o conhecimento matemático, a construção de um ecossistema que garanta altos níveis de segurança e confidencialidade para os pacientes, a aquisição de dados saudáveis, as necessidades de expandir a associação médico-paciente-dados, a necessidade de colaboração e o gerenciamento de cuidados baseados em dados. Nesse contexto, o cardiologista-jóquei (ou médicos em geral) deve ser protagonista das mudanças e deve substituir um eventual medo das ferramentas por um maior envolvimento, com o objetivo de gerar valor para o cuidado. É importante ter em mente os possíveis desafios e obstáculos a serem superados e manter um engajamento e senso crítico na busca de soluções: “quem corre é o cavalo, você precisa ser o jóquei”.
