# Supplementary material for: Inteligência Artificial em Cardiologia: Conceitos, Ferramentas e Desafios – “Quem Corre é o Cavalo, Você Precisa ser o Jóquei”
Source: Arq Bras Cardiol. 2020 May 12;114(4):718–25. [Article in Portuguese] doi: 10.36660/abc.20180431 (PMC9744354; doi:10.36660/abc.20180431)
Supplement: Translation

# Translation: Artificial Intelligence in Cardiology: Concepts, Tools and Challenges - “The Horse is the One Who Runs, You Must Be the Jockey”

**Authors:** Erito Marques de Souza, Fernando de Amorim Fernandes, Celine Lacerda de Abreu Soares, Flavio Luiz Seixas, Alair Augusto Sarmet M.D. dos Santos, Ronaldo Altenburg Gismondi, Evandro Tinoco Mesquita, Claudio Tinoco Mesquita

**Affiliations:** 1 Brazil Universidade Federal Fluminense, Niterói, RJ – Brazil; 2 Brazil Universidade Federal Rural do Rio de Janeiro - Departamento de Tecnologias e Linguagens, Nova Iguaçu, RJ – Brazil

**Keywords:** Artificial Intelligence/trends, Computer Systems/trends, Machine Learning/trends, Cardiovascular Diseases, Clinical Decision-Making

## Abstract

The recent advances at hardware level and the increasing requirement of personalization of care associated with the urgent needs of value creation for the patients has helped Artificial Intelligence (AI) to promote a significant paradigm shift in the most diverse areas of medical knowledge, particularly in Cardiology, for its ability to support decision-making and improve diagnostic and prognostic performance. In this context, the present work does a non-systematic review of the main papers published on AI in Cardiology, focusing on its main applications, potential impacts and challenges.

## Introduction

A person’s everyday life necessitates a huge amount of knowledge about the world and the volume of data in health grows exponentially throughout the world.^1^ On the other hand, biomedical knowledge is always expanding in an active and dynamic way and cannot be processed or stored by a single human brain. This situation makes it very difficult for the contemporary physician to keep up-to-date with such a broad spectrum of new data and findings, as well as to use such information easily and in a timely manner.^2^ Adding to this framework are the significant burnout rates among health professionals^3 , 4^ and the important impact of medical errors - which in the United States represent the third leading cause of death.^5^ This panorama brings with it the need to reorganize the productive structure of health services, associated with various challenges and new perspectives. Given that the current health system is generally unproductive and/or expensive, it is imperative to develop alternative and innovative strategies. The central focus for achieving this goal should be to increase the value for the patient – outcomes reached per dollar spent – so that good outcomes, efficiently obtained, are a target to be pursued.^6^

Besides, the recent advances at hardware level related to parallel processing, the existence of several machine-learning methods and the huge amount of annotated data contributed for artificial intelligence (AI) to promote a significant paradigm shift in the most diverse areas of medical knowledge and, particularly in Cardiology, for its ability to support decision-making that can improve diagnostic and prognostic performance. These impacts ought to be evaluated from the perspective of patient safety, personalization of care, value creation for the patients, within a scope of technological surveillance – that gradually consolidates AI as fundamental for a medical practice of excellence.^7 - 11^

This scenario makes AI, given its importance, be considered by many as the new electricity. The main journals in cardiology have published reviews in this area and the number of articles on the subject follows a growing trend, as shown in Figure 1 – this behavior is also seen in other medical specialties, such as Neurology. Therefore, the present work performs a non-systematic review of the main papers published on AI in Cardiology, focusing on its main applications, potential impacts and challenges. The next section presents the conceptual fundamentals on the topic, followed by a discussion on why cardiology needs AI and its main tools. Finally, the main challenges, perspectives and conclusions are presented.

**Figure 1:** – *Evolution of the number of works relating (Artificial Intelligence or Machine Learning) and Cardiology. Source: Pubmed. Accessed on 12/15/2018. Mesh Words: Cardiology and Machine Learning.*

### What is artificial intelligence?

The term AI was used for the first time at the Dartmouth Conference in 1956.^12^ Nevertheless, the possibility of machines being able to simulate human behavior and actually think was raised earlier by Alan Turing in 1950, who developed a test in order to differentiate humans from machines – thus named Turing test.^13^

Basically, AI is the product of the combination of sophisticated mathematical models and computation, which allows the development of complex algorithms capable of emulating human intelligence. All this process starts with the construction of a database representative of the problem that one wishes to study – adequately collected and processed– called healthy data. This step is of fundamental importance, as the algorithms will probably not perform well if this prerequisite is not obtained: " *garbage in, garbage out* ".

The nature of these data is quite varied, ranging from socio-environmental, clinical-laboratory, omic-data (e.g., metabolome, proteome, epigenome, lipidome) to information on red, green and blue intensities (RGB system) of each pixel that composes an image, for example. Equally diversified sources of such data include those obtained from electronic medical records or even wearable devices. In this context, the term Big Data is used to describe a huge collection of data for which traditional methods of analysis are unsuccessful in analyzing, searching, interpreting and storing.^9^

We highlight the use of these tools in problems of classification, regression, and clusterization. After obtaining healthy data and building the database, it is important to evaluate which mathematical models of AI are most appropriate for the problem that one wishes to solve. Then, the chosen models must be implemented using some programming language. A combination of models can also be useful. The results obtained by the algorithm should be analyzed in terms of both the coherence and suitability. These steps are summarized in Figure 2 .

**Figure 2:** – *Main Illustration.*

### Why does cardiology need artificial intelligence?

The development of AI algorithms has the advantage of not requiring many assumptions in relation to underlying data.^8^ Another point is that the nature of these mathematical-computational models allows, from observational data, a high level of evidence due to its high performance, which certainly represents a significant paradigm shift in evidence-based medicine. It should be noted that traditional clinical trials are generally slow, expensive, time-consuming, and limited in size.^14^ In addition, when the database is fed with more (healthy) data, in general, there is an improvement in the performance of the algorithms – which allows the studies to have a continuous character over time.

This new archetype can guide the allocation of scarce resources in the health area and facilitate the efficient and accurate identification of decisions that favor the individualization of care based on the flow of information that emerge from an integrated and complex ecosystem: it is a precision medicine.^15 , 16^ Therefore, it can be inferred that the practice of the cardiovascular sciences will have significant impacts, which will translate into a personalized approach and improved outcomes.

### Basic concepts in artificial intelligence

A generic database can be arranged in a matrix of rows and columns. Each line denotes an element from a set of objects to be evaluated according to the same features. Each column, in turn, expresses the values of a given attribute for the various rows in the database and each line represents a lesson to be learned by the mathematical-computational model. In this way, the term Machine Learning (ML) brings with it a possibility of " *learning* " from a set of lessons. The term AI is often used interchangeably with the term ML. However, ML is a subset of AI algorithms related the ability of learn from a large amount of data. AI is wider and encompass performing tasks that are normally related to human intelligence such as pattern recognition, problem solving, understanding language or recognizing objects and sounds.^17^

It is often said that the types of learning can be:

- **Supervised:** when the algorithm receives information about each lesson as well as the labels associated with it, having an important role in relation to the prediction. For example, if it is desired to predict whether a patient is more susceptible to cough with the use of angiotensin-converting enzyme inhibitors, analysis should be performed based on a healthy database containing a group of patients that showed such a reaction and another group in which this fact was not observed.
- **Unsupervised:** when the lesson labels are not provided *a priori* , it is up to the algorithm to find hidden structures in the database. A hypothetical example is the clusterization of a database of patients with hypertrophic cardiomyopathy according to imaging findings.
- **Reinforcement:** inspired by behavioral biology, it is a kind of reward-based learning.^18 , 19^

Another important concept is that of cognitive computing. It can be understood as a set of self-learning systems intended to imitate the human thought process based on the use of ML tools, pattern recognition and natural language processing.^9^ IBM Watson is an example of cognitive computing in the medical field.^20 , 21^

### Some artificial intelligence tools and applications

Currently, there is a multiplicity of models of ML each of them with diverse particularities, varied uses and limitations. The applications of some of these models in Cardiology are explained in the following paragraphs, while a brief description of each of them and their type is shown in Table 1 .

**Table 1:** – Brief description and classification of the main ML tools

Tool	Description	Learning
SVM	It is useful for two-group classification problems. The idea is to find a function called hyperplane from the resolution of a linear system built from the various lessons of the training subset. ^40^ This hyperplane is used to cluster the lessons of the test subset into two disjoint groups.	Supervised
NB	It was inspired in the studies of the reverend Bayes on conditional probability. ^41^ These probabilities are used to identify the category (out of a total n possible) that a particular lesson belongs to. ^42^	Supervised
KNN	It is said that a vector norm is a mathematical function, which satisfies specific properties, and associates a vector with a value greater than or equal to zero. ^43^ The norm of the difference between two vectors is the distance between them. The KNN uses a norm to calculate the distance between all the vectors (lessons) that make up the database. Then, for each vector of the database, the k vectors closest to it are determined. The inclusion in a given group is obtained from a majority voting system among the neighbors. ^44^^,^^45^	Supervised
AG	Algorithms inspired by the biological evolution of species, in which each possible candidate to solve the problem is modeled as a chromosome consisting of a set of genes, which during the execution of the algorithm undergoes operations of crossing-over and mutation in order to obtain better solutions than the current ones. ^46^ This way, they allow a database to be separated, for example, into two distinct groups – which have or do not have a particular characteristic.	Supervised
RF	This method is based on the construction of several decision trees. The first step is to get several random samples (with reposition) of lessons to build other databases, a process that is called bootstrapping. Each of these new databases will give rise to a decision tree, which is obtained iteratively, from a subset of variables (features). After the construction of all trees, a new lesson in the database should be allocated to the group that has the largest number of decision trees, showing that it belongs to this group (majority of votes). ^47^^,^^48^	Supervised
K-means	It allows partitioning a database into k groups with similar characteristics. To do so, it is necessary to update, in an iterative way, a set of vectors, called reference centroids of each group and to calculate the distance of each lesson to each one. A lesson is always allocated to the centroid for which it has the shortest distance. The elbow chart is generally used to determine the ideal number of groups to separate from the database. ^49^	Unsupervised
ANN	Inspired in biological nerve systems, a structure called a graph - a set of nodes and edges - is used in which nodes are layered and connected by valued edges, which represent a weight assigned to a given connection. The idea is that from a set of inputs, these weights are used properly to produce an output. Several architectures have been proposed for neural networks, from simpler ones such as the perceptron, to more sophisticated ones, such as the radial basis function, convolutional networks and deep learning. In deep learning, in addition to the input and output layers, there are hidden layers that increase significantly the number of weights to be updated and often require huge computational efforts. Convolutional network is a type of deep leaning inspired in visual cortex of animals that have an important role in image analysis. Autoencoders and Kohonen neural networks are examples of unsupervised learning. ^1^^,^^7^^,^^50^^-^^52^	Unsupervised or Supervised
GB	It is a tree-based method that uses gradient, vectors related to the direction of maximum increase in a math function, to produce sequential decision trees to be combined to brush up on the prediction. Variants of this approach include Stochastic Gradient Descent that incorporates a random subsampling to GB. ^53^^,^^54^	Supervised

- **Support Vector Machine (SVM):** used by Samad et al.,^22^ to predict with success the deterioration of ventricular function in patients with repaired tetralogy of Fallot from a database of 153 patients with clinical, electrocardiographic and cardiac magnetic resonance imaging data. In relation to predicting any deterioration (minor or major) vs. no deterioration, the mean area under the curve (AUC) was 0.82 ± 0.06.^22^ Berikol et al.^23^ used clinical, laboratory (troponin I and CK-MB levels), ECG, and echocardiographic data from 228 patients who presented at the emergency department with chest pain for classification regarding the presence or absence of Acute Coronary Syndrome. Accuracy, sensitivity and specificity were, respectively, 99.19, 98.22 and 100%.^23^ Betancur et al.^24^ also used SVM to more precisely define mitral valve plane (VP) positioning during left ventricular segmentation in Single-Photon Emission Computed Tomography (SPECT) exams. Images of 392 patients were analyzed and the good results obtained were compatible with the opinion of experts in the area – AUC: 0.82 [0.74-0.9] for regional detection of obstructive stenosis and ischemic total perfusion deficit areas.^24^
- **Naive Bayes (NB):** Paredes et al.,^25^ used an NB fusion and genetic algorithm to predict the risk of occurrence of cardiovascular events (e.g., hospitalization or death) based on data from 559 Acute Coronary Syndrome-Non-ST Segment Myocardial Infarction (ACS-NSTEMI) patients. Sensitivity and specificity were, respectively, 79.8, 83.8.^25^
- **K-nearest neighbors (KNN):** Al-Mallah et al.^26^ compared the prediction of all-cause mortality in 10 years between the classical logistic regression model and the KNN, considering a database of 34,212 patients with clinical information and information obtained after the treadmill test using the standard protocol of Bruce.^26^ The results obtained by this ML tool showed a sensitivity of 87.4% and specificity of 97.2%, better than the predictive performance of the traditional Atherosclerosis Cardiovascular Disease Risk Score (ASCVD).
- **Genetic algorithms (GA):** Smisek et al.^27^ developed a wearable device to detect arrhythmias from the information record of a single-lead electrocardiogram. The data were analyzed from a combination of the (SVM), decision tree and threshold-based rules. Genetic algorithms were used to select the most appropriate characteristics to be used in the work. In relation to the detection of atrial fibrillation, an F1 score (harmonic mean of positive predictive value and sensitivity) of 0.81 was obtained.^27^ Stuckey et al.,^28^ used the Cardiac Phase Space Tomography Analysis - a pioneering method that dispenses with the use of radiation and contrast, as well as performing exercises or pharmacological stress - combined with ML models (e.g., genetic algorithms) to analyze the thoracic phase signals. In this study, the authors used this tool to evaluate patients with coronary disease and chest pain who were referred by the physician for angiography. 606 patients were studied, and the results showed sensitivity of 92%, specificity of 62% and predictive value of 96% for coronary disease.^28^
- **Random Forests (RF):** Samad et al.,^29^ analyzed a database consisting of clinical and electrocardiographic variables to evaluate survival in 10 different periods of time (ranging from 6 to 60 months), considering a total of 171,510 patients. RF was used, with excellent results, better than those obtained through traditional scores such as the Framingham risk score and ACC/AHA guideline score. The area under the curve (AUC) was superior to 0.82.^29^ Ambale-Venkatesh et al.^30^ used information from noninvasive tests, questionnaires, biomarkers and imaging tests from 6,814 patients to construct 739 variables (features) in order to apply a variant of RF – called survivor random forests^31^ – for predicting cardiovascular events (all-cause death, stroke, all cardiovascular disease, coronary heart disease, atrial fibrillation and heart failure), having performed better than established risk scores, e.g., MESA-CHD, AHA/ASCVD and Framingham, with increased prediction accuracy (decreased Brier score by 10%-25%).^30 , 31^
- **K-means:** Cikes et al.^32^ used a database consisting of clinical variables and echocardiographic parameters for which two models of ML, Kmeans and Multiple Kernel Learning were applied, in order to categorize the patients into mutually exclusive groups to evaluate the response to resynchronization therapy cardiac. A total of 1,106 patients were analyzed and four disjoint groups were identified, two of them with the best response to therapy.^32^
- **Artificial Neural Networks (ANN):** Kwon et al.,^33^ in a multicenter study of 52,131 patients, constructed a deep learning-based early warning system capable of predicting the occurrence of cardiac arrest in a hospital. The model showed high performance when compared to traditional track-and-trigger systems. The area under the curve was 0.82.^33^ Rubin et al.,^34^ had promising preliminary results with the use of neural networks with convolutional architecture to evaluate electrocardiographic signs and to classify them in atrial fibrillation, sinus rhythm (normal) or noise – the F1 score achieved was 0.82.^34^ Zhang et al.^35^ also used convolutional neural networks to analyze a database with 14,035 echocardiographic exams to detect the presence of diseases such as hypertrophic cardiomyopathy, cardiac amyloidosis and pulmonary arterial hypertension with a high performance: C statistics were respectively, 0.93, 0.87, and 0.85.^35^ Nakajima et al.^36^ used an ANN to evaluate the presence of coronary disease after performing myocardial scintigraphy. Results were obtained with high accuracy and superior performance to the traditional scores used. For example, the AUC for patients with old myocardial infarction based on defects in rest stage was 0.97.^36^
- **Gradient Boosting (GB):** Mortazavi et al.^37^ used GB for prediction of risk of bleeding after percutaneous coronary intervention and demonstrated that these tools can help to identify patients who would benefit from strategies aiming to reduce the bleeding risk. A total of 3,316,465 procedures were analyzed and a C statistic of 0.82 was obtained.^37^ Hernesniemi et al.,^38^ also proposed a GB to predict mortality in acute coronary syndrome, analyzing 9,066 consecutive patients. The AUC was 0.89 and the model performed better than GRACE traditional score.^38^

It is important to note that when using any ML model, one should keep in mind a major problem that may arise, called overfitting. It occurs when a model describes the examples very well (training subset) and performs poorly when applied to other instances of the same phenomenon.^39^ In addition, it is worth saying that there is no theoretical result that ensures that any of the AI algorithms is better than the others in any application. Thus, this choice depends on several variables, such as the nature of the problem under analysis, the time and resources available to solve the problem. The combination of techniques generating hybrid models can also be of great value. On the other hand, the use of tools for parallel processing, such as the Graphic Processing Unit (GPU), has been of great value in improving the performance of ML models, especially in relation to computational time needed to run them.

### Challenges and future prospects

As previously highlighted, AI applications in cardiology have increased greatly in recent years and their growth potential is enormous. However, this scenario brings with it the need to overcome some challenges, such as: ethical limits of use (misuse), improvement of mathematical knowledge, acquisition of healthy data, development of security, need for collaboration, attention to errors and data-based care. All of this is discussed below and is summarized in Figure 2 .

- **Challenge 1 – ethical limits of use (misuse):** like all disruptive technology, the limits of ethics need to be rethought and widely discussed. ML algorithms can be misused and misleading. As an example, a work of great repercussion was published by Wang and Kosinski (2018). The authors used deep learning and obtained expressive results in the prediction of whether an individual is gay or not from a database of images of the study participants’ faces.^55^ Similarly, the same AI algorithms can be used to detect, for example, whether or not a patient will develop atrial fibrillation or any future cardiomyopathy. Could this information be used by companies to increase the amounts of their health plans or even deny membership to the plan due to a high cost? What if it is detected that a baby will be born with congenital heart disease due to the analysis of the genetic, clinical-laboratory and image (or other) data of its parents? This could open space for a kind of neoeugenia. This debate has gained an additional emphasis with the emergence of the CRISP-Cas9 technique, which allows DNA editing.^56^ In this context, by stimulating a debate with society on the subject, transparency and regulation are fundamental pillars to be preserved.
- **Challenge 2** **–** **improve math knowledge:** the advent of this new kind of unbelievable human being ( *Homo incredibile* ), which supports its decisions in data, carries with it the fundamental role of mathematics and computation in this currently ongoing revolution. This revolution will bring unimaginable possibilities in medical practice, such as the construction of quality phenomappings - ML models developed with the aim of clustering patients in function of their large mass of phenotypic characteristics in order to facilitate the decision-making process.^57^ Thus, it is necessary that these competencies are stimulated early, mainly with a focus on solving problems related to the reality for which one wishes to promote improvements. This will certainly be reflected in a need to reformulate the cardiovascular contents (and why not say, medical content in general) of undergraduate and postgraduate courses in Medicine: a passive or merely expositive education, with an extensive load and that prioritizes the capacity of the student's memory seems, more and more, to be inadequate, as one realizes that Medicine must be a space for creativity and value generation.
- **Challenge 3 –** get healthy data: the use of healthy data is of fundamental value for the success of the algorithms. Thus, it is required that health units encourage their health professionals regarding the thoroughness at the level of data filling/obtaining as well as maintaining any data sources, from forms, electronic medical records, image data or even unconventional data, such as those obtained by Medina et al.^58^ - who developed a successful Online Social Networks Health tool in which the patient himself anonymously inserts health monitoring information, including physiological data, daily activities, emotional states, and interaction with others patients.^58^ Therefore, data management becomes as important as other routine behaviors in evidence-based medicine, such as proper handwashing or even the use of a defibrillator during cardiac arrest. In this way, the formation of multidisciplinary data teams and the constant training of the teams assume a primordial role. It is noteworthy that much of the slowness and difficulty that some health units have in using ML models is tied to absent or incipient healthy data.
- **Challenge 4 –** get security: the advent of these tools brings with it a fundamental concern with data security, to a level never before experienced, as access to such data by unauthorized persons can lead to catastrophic consequences for both health institutions and the patients. The creation of a security team plays an important role in this new process. The General Data Protection Regulation represents an advance in this direction. Blockchain and its variants are important tools that can improve security substantially.
- **Challenge 5 –** need for collaboration (the African proverb): there is an African proverb that says, " *if you want to go fast go by yourself, but if you want to go far go with many* ". This applies a lot to this data environment: collaboration between institutions allows the construction of huge healthy databases (Big Data), which tends to favor the performance of ML algorithms.
- **Challenge 6 –** dealing with errors: one important issue concerns the errors of AI models. It is inadequate to believe that such models are error-free. It may, for example, be the result of overfitting or occurring by using unhealthy data - which make the results unreliable. However, the practice has shown high performance in several applications. These models are probabilistic, and it is always desirable that their errors be minimal. This scenario has clinical implications, for example, an AI model that predicts with 99% probability that a patient has a greater propensity than the general population to have cardiac myocarditis or amyloidosis. There is a probability, although small, that this will not occur, and that the procedure adopted by the cardiologist is inadequate. In that case, the question is who can be held accountable in these cases? Is it appropriate? Should the patient sign a consent form in these cases? Certainly, the solution includes robust regulation of the use of these tools and strengthening of a new type of relationship: physician-patient-data.
- **Challenge 7 –** data-based care management: while ML's tools follow an inexorable path, on the other hand, several healthcare professionals remain fearful about these tools because of its possible ability to replace physicians in their tasks. However, when the history of Medicine is remembered, it is worth mentioning, for example, that the appearance of automated machines to perform the whole blood count did not replace the hematologist, but rather resulted in a greater speed of the work process and allowed the professional to be able to act in other important issues in the specialty.

The central idea is to provide better support for decision-making, including better performance. It is data-driven care management with a high dynamism and constant updating - which will promote greater personalization of care^59^ and a real-time evaluation of the experience of the health system users, aiming at generating value for the patient. In this context, the mechanical tasks will be substitutable and a diversity of new tasks will be included into the routine of the cardiologist of precision, from the adequate construction of the databases to the critical reflection on the results obtained by the mathematical-computational models, as well as the development of an adequate physician-patient-data relationship. Therefore, there is a migration of human skills as well as the expansion of their capabilities from the emergence of new tools, which should be part of the technical arsenal of the 21^st^century cardiologist. This panorama allows us to compare ML models to a horse and doctors to jockeys: " *the horse is the one who runs , you must be the jockey* ".

## Conclusions

AI, in fact, has been shown to be a fundamental tool for the clinical practice of current cardiology. Several applications have been successfully performed and have allowed significant improvements from a diagnostic and therapeutic point of view and in relation to personalized care. To be able to use such tools, it is imperative that healthy data be used, which certainly implies a new design in the modus operandi of many health services. The nature of these data is varied and includes new sources, such as wearable devices and omic-data. On the other hand, this new digital ecosystem requires an acquisition of knowledge not traditionally found in regular medical courses. Therefore, a curricular redesign is required and ought to be object of a profound debate and specific actions.

On the other hand, the entire panacea brought by AI is not free from challenges such as: the ethics limits of its use, the necessity of improving math knowledge, the building of an ecosystem that ensure high levels of security and confidentiality for the patients, the acquisition of healthy data, the needs of expand the physician-patient-data association, the necessity of collaboration and the data-based care management. In this context, the cardiologist-jockey (or physicians in general) must be a protagonist of changes and has to replace an eventual fear of the tools by a greater involvement with the objective of generating value for the care. It is important to keep in mind possible challenges and obstacles to be overcome and to maintain an engagement and critical sense in the search for solutions: "the horse is the one who runs, you must be the jockey".
